# Supplementary material for: A Critical View of the Application of the APEX Software (Aqueous Photochemistry of Environmentally-Occurring Xenobiotics) to Predict Photoreaction Kinetics in Surface Freshwaters
Source: Molecules. 2019 Dec 18;25(1):9. doi: 10.3390/molecules25010009 (PMC7017383; doi:10.3390/molecules25010009)
Supplement: Supplementary file 1 [file molecules-25-00009-s001.zip › 3.2.4_gcc-4.4.0.zip!/3.2.4_gcc-4.4.0/3.2.4_gcc-4.4.0/doc/octave/html/interpreter/Output-Conversion-for-Matrices.html]

Output Conversion for Matrices - Untitled


Next: Output Conversion Syntax,
Previous: Formatted Output,
Up: C-Style I/O Functions


---

#### 14.2.5 Output Conversion for Matrices

When given a matrix value, Octave's formatted output functions cycle
through the format template until all the values in the matrix have been
printed. For example,

```
     printf ("%4.2f %10.2e %8.4g\n", hilb (3));
     
          -| 1.00   5.00e-01   0.3333
          -| 0.50   3.33e-01     0.25
          -| 0.33   2.50e-01      0.2
```

If more than one value is to be printed in a single call, the output
functions do not return to the beginning of the format template when
moving on from one value to the next. This can lead to confusing output
if the number of elements in the matrices are not exact multiples of the
number of conversions in the format template. For example,

```
     printf ("%4.2f %10.2e %8.4g\n", [1, 2], [3, 4]);
     
          -| 1.00   2.00e+00        3
          -| 4.00
```

If this is not what you want, use a series of calls instead of just one.
